# Suspected human anthrax outbreak investigation in a tribal village of Koraput, India, 2021

**DOI:** 10.1002/puh2.125

**Published:** 2023-12-04

**Authors:** Jyoti Shandilya, Debaprasad Parai, Hari Ram Choudhary, Jaya Singh Kshatri, Biren Kumar Padhy, Padma Mohan Pradhan, Deepika Saket, Annalisha Peter, Matrujyoti Pattnaik, Arun Kumar Padhi, Sanghamitra Pati, Debdutta Bhattacharya

**Affiliations:** ^1^ Department of Microbiology ICMR‐Regional Medical Research Centre (Department of Health Research Ministry of Health & Family Welfare) Government of India Bhubaneswar India; ^2^ Department of Health and Family Welfare Office of the Chief District Medical Officer Koraput Government of Odisha Koraput India

**Keywords:** anthrax, endemic, outbreak, outbreak investigation, rural

## Abstract

**Background:**

Odisha is a state in India endemic to anthrax disease with frequent reports of suspected animal cases. A suspected outbreak of anthrax in humans was reported on 24 October 2021 at Tukum village in Koraput district of Odisha, India after a bullock was found dead and consumed by the locals on 17 October 2021.

**Methods:**

This extended outbreak investigation was carried out through house‐to‐house active surveillance from 24 October to 2 November 2021 in the Koraput district. Eschar skin swabs from wounds were collected and processed at District Public Health Laboratory, Koraput, and analyzed in Indian Council of Medical Research‐Regional Medical Research Centre, Bhubaneswar for molecular confirmation. Samples from bone, soil, and dried meat were collected from the contaminated sites and were transported to Animal Diseases Research Institute, Cuttack for confirmation.

**Results:**

Four suspected cases of human anthrax were identified who had handled and consumed dead bullock meat, among which one human had died later. The attack rate of the persons at risk in the village was calculated to be 1.23%. However, no *Bacillus anthracis* were identified in human swab samples when tested in real‐time polymerase chain reaction. Samples collected from contaminated sites were confirmed to have anthrax bacilli.

**Conclusion:**

Investigation revealed that a suspected anthrax cluster outbreak was due to butchering/de‐skinning and consumption of the anthrax‐infected dead animal. The presence of bacilli in human samples could not be confirmed due to the intake of antibiotics before the collection of sample. This finding highlighted the importance of sample collection at a suitable time and a possible need for one health approach for better coordination among the different responsible departments.

## Graphical abstract

A suspected human anthrax outbreak was notified in the Koraput district of Odisha, India in October 2021. Routine surveillance, timely vaccination of livestock, and proper disposal of livestock carcasses are the most efficient ways of preventing and controlling anthrax infection in domestic herds.

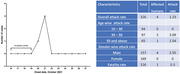

## INTRODUCTION

Anthrax is a zoonotic disease caused by *Bacillus anthracis* that affects herbivorous animals [1]. This organism naturally exists as a spore form in soil, infects domestic, and wild animals during grazing and can be transmitted to humans when in close proximity or contact [2]. Anthrax spores from carcasses of dead animals contaminate the soil and persist for more than decades. According to the National Animal Diseases Referral Expert System, anthrax is among the top 10 diseases reported in India and one of the major causes of death in livestock. As India has a variety of agroclimatic zones, the occurrence of anthrax spores varies across the country [3, 4].

Anthrax continues to affect and is endemic in multiple global regions like Mediterranean countries, a few parts of Canada and the United States, some central and south American and central Asian countries, a few African countries, and western China [5]. In India, anthrax is enzootic in Andhra Pradesh, Jammu and Kashmir, Tamil Nadu, Odisha, and Karnataka [6]. It primarily affects communities living near forest areas, especially in Eastern states like Odisha where outbreaks are mostly documented and reported [7]. In the last 15 years, 14 out of 30 districts in Odisha have witnessed anthrax outbreaks, affecting at least 1208 people, 436 of whom have died [8].

There are three forms of anthrax–cutaneous, gastrointestinal, and inhalational. In the majority of patients, cutaneous anthrax accounts for 95%, and the remaining other type (gastrointestinal and pulmonary anthrax) accounts for 5% of cases. The most common cause of cutaneous anthrax in humans is due to direct contact with infected animals or animal products. Lack of coordination between animal and human health sectors often delayed the early detection, laboratory confirmation, containment, and prevention of anthrax outbreaks [9].

Koraput is a tribal district in Odisha where animal anthrax is endemic and livestock vaccination rates are low. On 24 October 2021, the Koraput District Integrated Disease Surveillance Program (IDSP) unit was notified of human cases with skin ulcers for a clinically suspected anthrax case. All human anthrax cases were diagnosed based on the unified case definitions issued by the IDSP and the National Health Mission, India, in 2018 [10]. This study was conducted by the Indian Council of Medical Research‐Regional Medical Research Centre (ICMR‐RMRC), Bhubaneswar in collaboration with the district Rapid Response Team (RRT) to investigate the source of suspected anthrax outbreak, determine its transmission mechanism, find the gaps, and implement control measures in that region.

## METHODS

### Study setting

**FIGURE 1:**
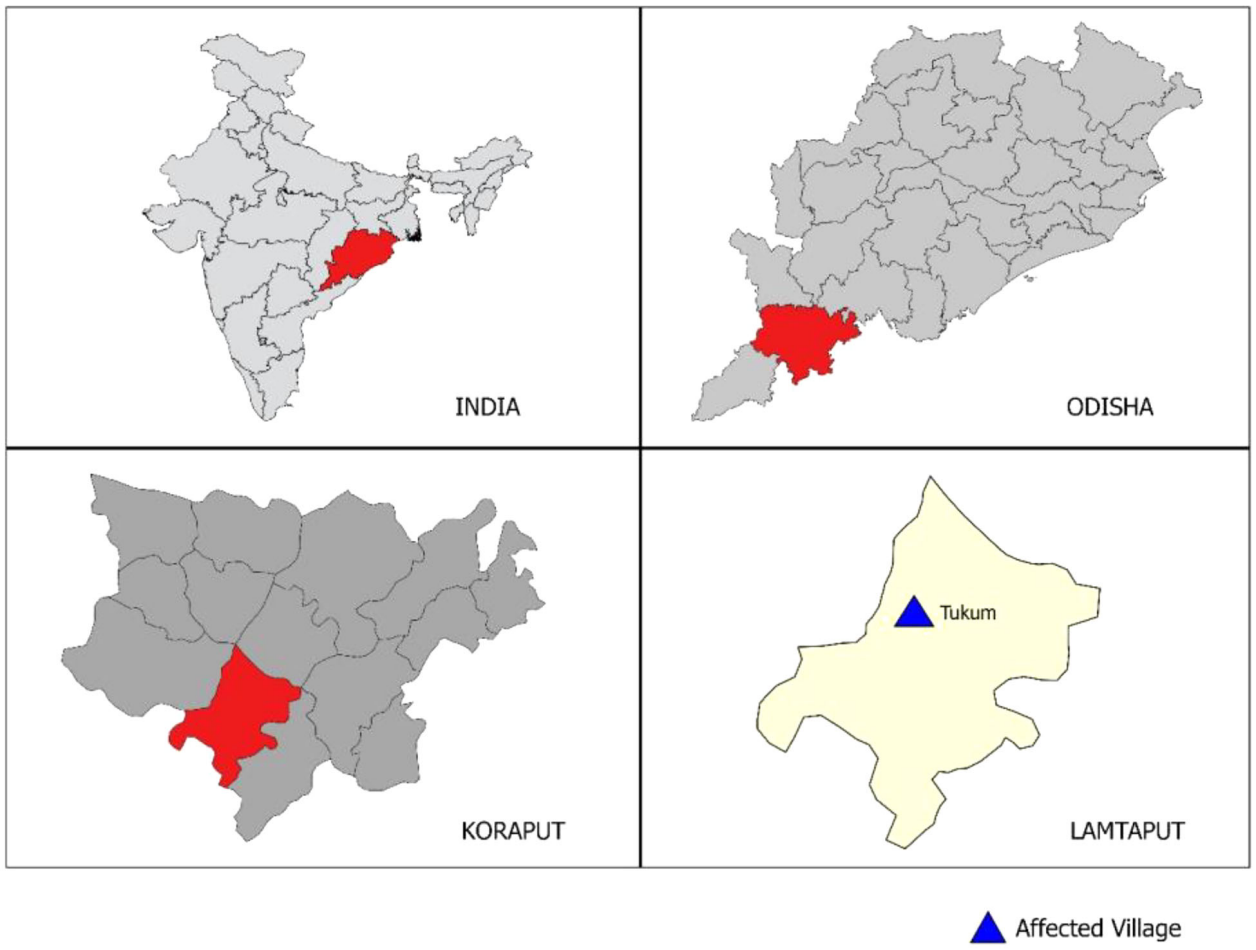
Study setting of anthrax outbreak occurred in October 2021, Koraput District.

This outbreak investigation was conducted in Tukum village of Jalahanjar GP (Gram panchayat; village administrative body) of Lamtaput block in Odisha, India after receiving reports of suspected cases from the IDSP unit (Figure 1). The investigation was carried out from 24 October 2021 to 2 November 2021. Based on the Census of India (2011) population data, the population of this village was 326.

### Case investigation and formation of response team

**FIGURE 2:**
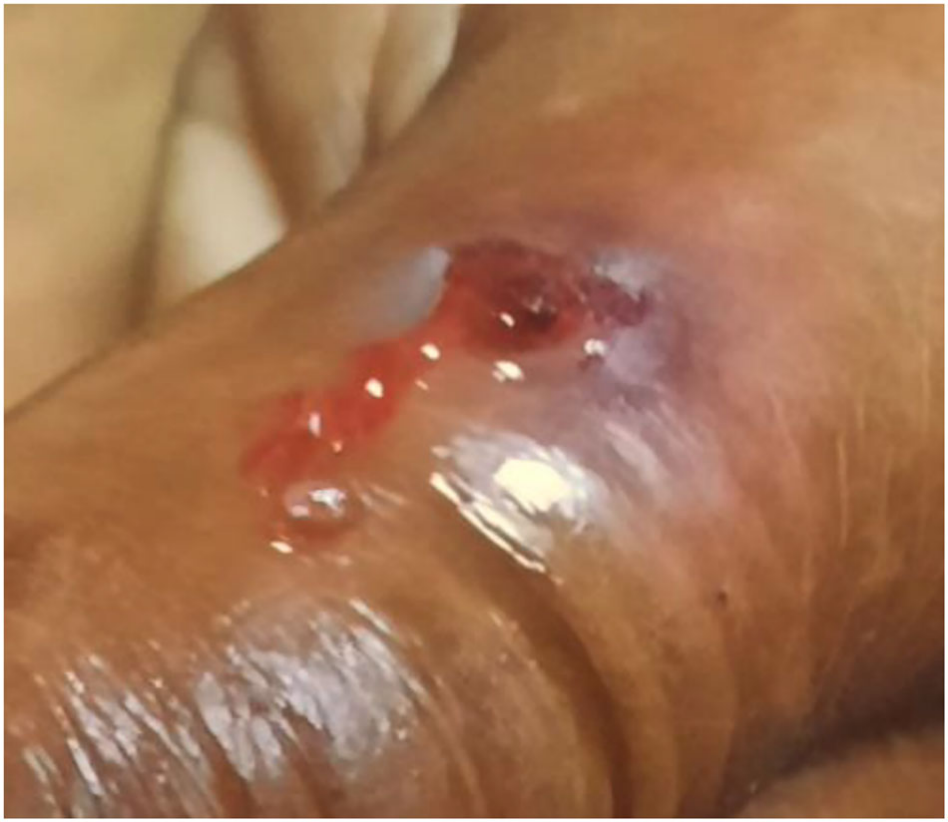
Ulcer on right index finger dorsal end as found in a suspected human case.

We defined a suspected cutaneous anthrax case as a painless skin lesion such as a vesicle, eschar, or ulcer with illness onset between 20 April and 30 October 2021 in the Lamtaput block of Koraput district. On 24 October 2021, four clinically suspected cutaneous anthrax cases were admitted either to the Community Health Centres (CHC), Lamtaput, or Saheed Laxman Nayak Medical College and Hospital, Koraput (SLNMCH), respectively, after the development of papule, vesicle, and pustule over the upper extremity (Figure 2). It was reported that all the cases came into contact with a dead bullock on 17 October 2021. A house‐to‐house survey was conducted immediately to investigate the source of the cases. The district RRT which constituted of a clinician, district microbiologist, district epidemiologist, and laboratory technician immediately reached the site and initiated containment, investigation, and surveillance.

### Trace‐forward and environmental investigation

House‐to‐house surveys were conducted with the support of the district Health department and Accredited Social Health Activist members in the village to know about their history of exposure to the dead bullock, if any. All the suspected anthrax cases were tracked by their exposure history and medical examination. Eschar skin swabs from the wound and 5 mL of blood were collected from all the suspected cases who developed signs by district RRT with the help of the SLNMCH team and were sent to the District Public Health laboratory for initial confirmation by Gram staining. All the livestock population data (cattle) along with the vaccination campaigns were reviewed after taking records from the district veterinary department.

### Laboratory investigation

Eschar skin swab samples were further sent to ICMR‐RMRC, Bhubaneswar to confirm the presence of *B. anthracis* by real‐time polymerase chain reaction (RT‐PCR). DNA was extracted using a commercial kit QIAmp DNA mini kit (Qiagen), and amplification was done targeting two genes: protective antigen gene (*pagA*) and capsule synthesis gene (*capC*) following a standard protocol [11]. Soil samples from the environment (where the dead animal was found) along with bones and dried meat samples were collected and sent to the Animal Disease Research Institute, Cuttack for the culture of specimens and respective RT‐PCR. The attack rate was calculated as the number of people who became ill due to anthrax divided by the number of people at risk for the illness.

## RESULTS

**FIGURE 3:**
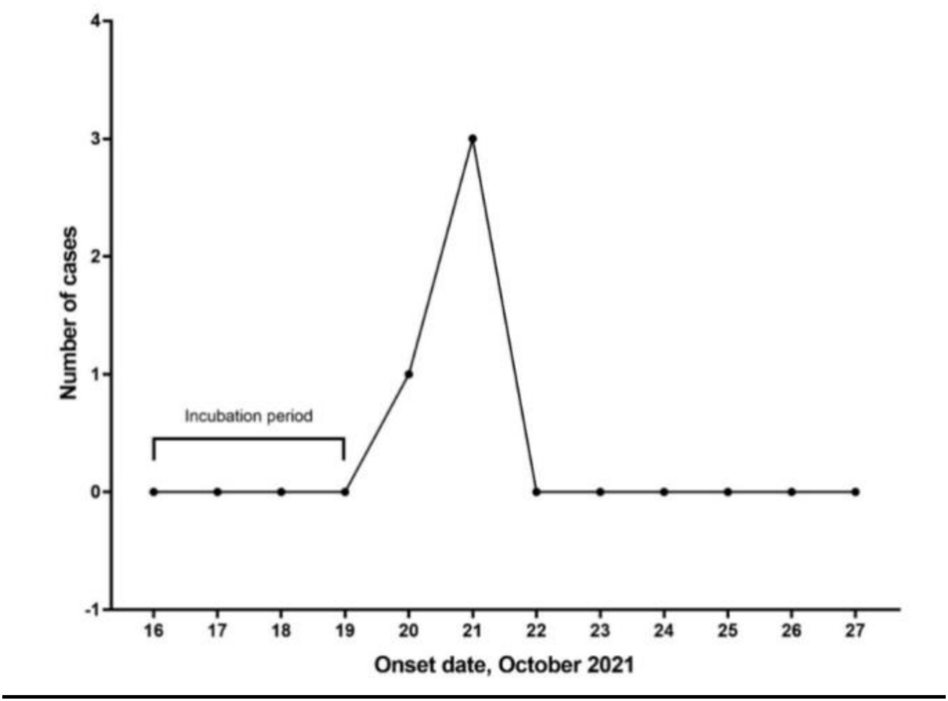
Epidemic curve of a suspected anthrax outbreak in Koraput, India, 2021.

During house‐to‐house surveys, it was recorded that the cattle died suddenly in the evening after returning from daily grazing. No history of clinical complications or behavioral abnormalities was noticed before the death. However, the owner found traces of very little amount of blood inside the nose after the death. Eventually, villagers slaughtered the dead bullock, and the meat was distributed among the community peoples as a part of social rituals. Two more cattle were reported later to have high fever and loss of appetite, and treated accordingly, and eventually, they were cured without any further casualties. A total of 60 people were exposed to the suspected source by direct contact either during handling or through the consumption of meat. Among those, 51 were involved only in the ingestion of the dead animal, and other 9 persons did both the butchering/de‐skinning and consumption of the same (Supporting Information Annexures 1 and 2). A total of four human cases developed skin lesions and were from that second category who handled and ate the dead meat. Symptoms started to develop among these individuals on 20 October 2021, and the incubation period ranged between 3 and 4 days (Figure 3). Median age of the respondents was 43 years, and the majority of them were females (53.33%) (Table 1). Around 1.67% of cases had developed ulcerative lesions, and 3.33% of cases had developed both pustules and papules/rash. All of the cases were referred either to CHC Lamtaput or SLNMCH for treatment. There was one death among four people admitted to the health facility. This patient presented with unequal pupils, seizures, convulsions, and high random sugar. He had consumed a high dose of alcohol for 2 days before he died, and his death was likely caused by raised intracranial pressures or encephalitis. The attack rate of the persons at risk in the village was calculated to be 1.23%. Age group of 30–50 years had an attack rate of 3.09%, and the ≥50 years age group had 1.54% for the same. Attack rate of males was 2.25%, whereas among females it was nil (Table 2). Fatality rate of the outbreak was calculated to be 0.3%. A very small number of Gram‐positive rods were detected by Gram staining although the RT‐PCR test could not confirm the presence of the specified genes of *B. anthracis* in human cutaneous swab samples. However, the culture of samples from contaminated soil, bone, and dried meat showed no growth in culture media but was RT‐PCR positive for *B. anthracis* as reported by the state veterinary department. Tukum village had not received any vaccination by that time as part of the district's annual livestock vaccination program. After the suspected outbreak incidence, 300 cattle out of 388 in that village were vaccinated on an emergency basis, and ring vaccinations were also done in the neighboring villages within a 5‐km radius, as well as in all the epicenters where anthrax had been endemic for the past 10 years.

**TABLE 1:** Sociodemographic characteristics of respondents involved in consumption of the meat from a bullock that died of suspected anthrax, Koraput, India, 2021.

Characteristics	*n*	%
Age (in years)		
10–30	17	28.33
30–50	21	35.00
50 and above	22	36.67
Sex		
Male	28	46.67
Female	32	53.33
Symptoms and signs		
Ulcerative lesion	1	1.67
Pustule	2	3.33
Papule and rash	2	3.33
No symptoms	56	93.33
Occupation		
Agriculture	19	31.67
Housewife	26	43.33
Unemployed	3	5.00
Student	12	20.00

**TABLE 2:** Attack and fatality rate calculations of persons at risk of suspected human anthrax outbreak, Koraput, India, 2021.

Characteristics	Total	Affected humans	Attack rate
Overall attack rate	326	4	1.23
**Age‐wise attack rate**			
10–30	94	0	0
30–50	97	3	3.09
50 and above	65	1	1.54
**Gender‐wise attack rate**			
Male	157	4	2.55
Female	169	0	0
**Fatality rate**	**326**	**1**	**0.3**

## DISCUSSION

Rapid diagnosis, isolation, antibiotic therapy as well as preventive measures, should be taken to reduce the disease progression and manage the outbreaks promptly and efficiently. In outbreaks recorded from Africa and Southeast Asia, sociocultural practices such as butchering, consuming, handling, and dumping of dead animals in the open places among animal handlers have contributed to anthrax outbreak transmission and progression [12]. Coordinated efforts are needed at the community level to promote the utilization of veterinary services and vaccination of livestock to protect the animal health and livelihoods of livestock owners, to promote proper disposal practices, and to avoid slaughtering sick animals for consumption to protect human health [13].

In this study, the suspected anthrax outbreak was reported mostly among males in the tribal community in the Koraput district of Odisha. We have found that de‐skinning of dead animals is a cultural practice among the tribal community in the affected village, and most males are involved in such practice which puts men at a higher risk of infection with cutaneous anthrax. So, focusing on and increasing public health education could be a primary measure to prevent and control anthrax in this district.

Similar suspected outbreaks of human anthrax cases were reported with multiple exposures from handling the carcass of a cow in the Kween district of Uganda in April 2018 [14]. In India, seven cases of suspected anthrax were found in West Bengal, all of which occurred after the slaughtering of animals [15]. Anthrax continues to be endemic in underdeveloped regions due to less awareness and food‐related practices among the tribal populations.

Our household investigation revealed a lack of awareness about anthrax and poor living conditions due to low socioeconomic status in Tukum. These conditions likely facilitated the zoonotic transmission from animals to humans, leading to the subsequent outbreak. As a part of outbreak response, control measures such as the disinfection of tube well platform drains and cattle shed of the village were undertaken. The veterinary department traced the contamination site and immediately destroyed the dead carcasses after suspected human cases were reported. Messages such as “strictly avoiding consumption of dead meat,” “not to handle sick animals without PPE,” and “safe disposal of the carcass” were delivered by health workers in collaboration with the veterinary department to increase awareness among the villagers. All the residents of Tukum village were requested to report or notify the local veterinarian or health workers in case of any suspected events.

We speculated this as an anthrax outbreak because of the positive RT‐PCR results for *B. anthracis* detected in the environmental samples and dead bullock residuals. However, the negative result of human samples might be due to the initiation of antibiotic treatment before the sample collection and henceforth we failed to reconfirm the human outbreak. During the investigation, we found that anthrax detection both in animal and human samples is limited due to a lack of event‐based surveillance or syndromic surveillance systems and proper diagnostic facilities at the district level in Koraput. Therefore, strengthening laboratory capability is crucial for the early detection of an outbreak at the district level. Moreover, as a part of outbreak response, interdepartmental coordination needs to be strengthened between human health and veterinary departments in the district which might be best translated by “One Health” approach. Therefore, we have now attempted to strengthen these gaps and made efforts to sensitize the stakeholders through various One Health interventions like active surveillance, animal vaccination, interdepartmental coordination, capacity building of various stakeholders via training, information education communication, and behavioral change communication to increase the awareness, knowledge, attitude, and practices of the community people toward anthrax.

Our outbreak investigation is subjected to a few limitations. The negative results of swab samples collected from the skin lesions of suspected cases might be due to antibiotic consumption before sample collection. However, despite this, we were able to trace the cases of those who had consumed and were involved in the butchering of the bullock that died due to anthrax. The village livestock inspector and healthcare workers had limited information available with regard to the previous history of anthrax in Tukum village and the status of livestock vaccination against anthrax. The unavailability of rapid antigen test kits in the Indian market could be another limitation of this investigation.

## CONCLUSION

This outbreak was confirmed by the positive RT‐PCR result by the veterinary department. However, any suspected human cases could not be confirmed further by the molecular diagnosis due to late reporting, delay in sample collection, and administration of antibiotics. Thus, our study highlighted the importance of timely sample collection for establishing any anthrax outbreak and to plan an effective management strategy. Routine surveillance, timely vaccination of livestock, and proper disposal of livestock carcasses are the most efficient ways of preventing and controlling anthrax infection in domestic herds, which eventually limits its transmission to humans. We advocate that the implementation of the One Health approach for better coordination among the veterinary, forest, and public health sectors is much needed for the rapid detection of zoonotic diseases like anthrax.

## AUTHOR CONTRIBUTIONS

Sanghamitra Pati, Jaya Singh Kshatri, Debdutta Bhattacharya, and Arun Kumar Padhi conceptualized and designed the study. The field activities and data collection were executed by Jaya Singh Kshatri, Hari Ram Choudhary, Biren Kumar Padhy, Padma Mohan Pradhan, Deepika Saket, Matrujyoti Pattnaik, Debaprasad Parai, and Arun Kumar Padhi did laboratory analysis of the samples collected for confirmation. Jaya Singh Kshatri, Debaprasad Parai, and Hari Ram Choudhary wrote the initial draft of the manuscript and reviewed it by Jaya Singh Kshatri, Arun Kumar Padhi, Debdutta Bhattacharya, and Sanghamitra Pati. All the authors have reviewed and approved the manuscript for publication.

## CONFLICT OF INTEREST STATEMENT

The authors have no conflicts of interest in any form.

## FUNDING INFORMATION

Indian Council of Medical Research, New Delhi (Grant Number: ZON/33/1/2018‐ECD‐II). The funding body was not involved in the design of the study and collection, analysis, and interpretation of data, or in writing the manuscript.

## ETHICS STATEMENT

The study was approved by the Institutional Human Ethics Committee of ICMR‐Regional Medical Research Centre, Bhubaneswar. All the methods were carried out per ICMR guidelines and regulations. Written informed consent was taken from respondents ≥18 years of age. An assent form was signed by the children (aged between 12 and 17 years) as well as a consent form was obtained from their parents or guardians. Data were not shared outside of the investigation team.

## Supporting information

Supporting Information
