# Supplementary material for: Suspected human anthrax outbreak investigation in a tribal village of Koraput, India, 2021
Source: Public Health Chall. 2023 Dec 4;2(4):e125. doi: 10.1002/puh2.125 (PMC12039731; doi:10.1002/puh2.125)
Supplement: Supplementary file 1 — Supporting Information [file PUH2-2-e125-s001.docx]

**Annexure-1. Line listing of the respondents who were engaged both in butchering and consumption**

| **Sl. No** | **Age** | **Sex** | **Mode of Exposure** | **Clinical Signs present** | **Date of Onset of signs and symptoms** | **Date of Diagnosis/treatment** | **Final Outcome** |
| --- | --- | --- | --- | --- | --- | --- | --- |
| 1 | 36 | M | Contact and Ingestion | No clinical signs | 20.10.21 | 23.10.21 | Death at Asha Kiran Hospital, Koraput |
| 2 | 42 | M | Contact and Ingestion | Ulcerative lesion on the finger | 21.10.21 | 23.10.21 | Discharged and follow up |
| 3 | 53 | M | Contact and Ingestion | Pustule, Vesicles over upper extremities | 21.10.21 | 23.10.21 | Discharged and follow up |
| 4 | 45 | M | Contact and Ingestion | Papule and Rash | 21.10.21 | 23.10.21 | Discharged and follow up |
| 5 | 35 | M | Contact and Ingestion | No symptoms | No symptoms | 23.10.21 | Not admitted |
| 6 | 43 | M | Contact and Ingestion | No symptoms | No symptoms | 23.10.21 | Not admitted |
| 7 | 32 | M | Contact and Ingestion | No symptoms | No symptoms | 23.10.21 | Not admitted |
| 8 | 49 | M | Contact and Ingestion | No symptoms | No symptoms | 23.10.21 | Not admitted |
| 9 | 54 | M | Contact and Ingestion | No symptoms | No symptoms | 23.10.21 | Not admitted |

**Annexure-2. Line listing of respondents who had consumed the dead bullock meat**

| **Sl. No** | **Date of house-to-house visits** | **Village** | **Sex** | **Age** | **Clinical signs and symptoms** | **Date of prophylaxis** | **Mode of exposure** |
| --- | --- | --- | --- | --- | --- | --- | --- |
| 1 | 24/10/21 | Tukum | M | 65 | None | 24/10/21 | \| Ingestion of carcass \| \| --- \| |
| 2 | 24/10/21 | Tukum | F | 61 | None | 24/10/21 | \| Ingestion of carcass \| \| --- \| |
| 3 | 24/10/21 | Tukum | F | 38 | None | 24/10/21 | \| Ingestion of carcass \| \| --- \| |
| 4 | 24/10/21 | Tukum | M | 45 | None | 24/10/21 | \| Ingestion of carcass \| \| --- \| |
| 5 | 24/10/21 | Tukum | M | 67 | None | 24/10/21 | \| Ingestion of carcass \| \| --- \| |
| 6 | 24/10/21 | Tukum | F | 65 | None | 24/10/21 | \| Ingestion of carcass \| \| --- \| |
| 7 | 24/10/21 | Tukum | M | 63 | None | 24/10/21 | \| Ingestion of carcass \| \| --- \| |
| 8 | 24/10/21 | Tukum | F | 61 | None | 24/10/21 | \| Ingestion of carcass \| \| --- \| |
| 9 | 24/10/21 | Tukum | M | 58 | None | 24/10/21 | Ingestion of carcass |
| 10 | 24/10/21 | Tukum | F | 55 | None | 24/10/21 | \| Ingestion of carcass \| \| --- \| |
| 11 | 24/10/21 | Tukum | F | 30 | None | 24/10/21 | \| Ingestion of carcass \| \| --- \| |
| 12 | 24/10/21 | Tukum | M | 60 | None | 24/10/21 | \| Ingestion of carcass \| \| --- \| |
| 14 | 24/10/21 | Tukum | F | 67 | None | 24/10/21 | \| Ingestion of carcass \| \| --- \| |
| 15 | 24/10/21 | Tukum | M | 57 | None | 24/10/21 | \| Ingestion of carcass \| \| --- \| |
| 16 | 24/10/21 | Tukum | M | 31 | None | 24/10/21 | \| Ingestion of carcass \| \| --- \| |
| 17 | 24/10/21 | Tukum | F | 25 | None | 24/10/21 | Ingestion of carcass |
| 18 | 24/10/21 | Tukum | F | 13 | None | 24/10/21 | \| Ingestion of carcass \| \| --- \| |
| 19 | 24/10/21 | Tukum | M | 52 | None | 24/10/21 | \| Ingestion of carcass \| \| --- \| |
| 20 | 24/10/21 | Tukum | F | 60 | None | 24/10/21 | \| Ingestion of carcass \| \| --- \| |
| 21 | 24/10/21 | Tukum | M | 45 | None | 24/10/21 | \| Ingestion of carcass \| \| --- \| |
| 22 | 24/10/21 | Tukum | F | 41 | None | 24/10/21 | \| Ingestion of carcass \| \| --- \| |
| 23 | 24/10/21 | Tukum | F | 12 | None | 24/10/21 | \| Ingestion of carcass \| \| --- \| |
| 24 | 24/10/21 | Tukum | F | 14 | None | 24/10/21 | \| Ingestion of carcass \| \| --- \| |
| 25 | 24/10/21 | Tukum | M | 27 | None | 24/10/21 | \| Ingestion of carcass \| \| --- \| |
| 26 | 24/10/21 | Tukum | F | 20 | None | 24/10/21 | \| Ingestion of carcass \| \| --- \| |
| 27 | 24/10/21 | Tukum | F | 12 | None | 24/10/21 | \| Ingestion of carcass \| \| --- \| |
| 28 | 24/10/21 | Tukum | F | 12 | None | 24/10/21 | \| Ingestion of carcass \| \| --- \| |
| 29 | 24/10/21 | Tukum | M | 18 | None | 24/10/21 | \| Ingestion of carcass \| \| --- \| |
| 30 | 24/10/21 | Tukum | M | 65 | None | 24/10/21 | \| Ingestion of carcass \| \| --- \| |
| 31 | 24/10/21 | Tukum | F | 63 | None | 24/10/21 | \| Ingestion of carcass \| \| --- \| |
| 32 | 24/10/21 | Tukum | F | 48 | None | 24/10/21 | \| Ingestion of carcass \| \| --- \| |
| 33 | 24/10/21 | Tukum | F | 45 | None | 24/10/21 | \| Ingestion of carcass \| \| --- \| |
| 34 | 24/10/21 | Tukum | F | 19 | None | 24/10/21 | \| Ingestion of carcass \| \| --- \| |
| 35 | 24/10/21 | Tukum | F | 16 | None | 24/10/21 | \| Ingestion of carcass \| \| --- \| |
| 36 | 24/10/21 | Tukum | M | 40 | None | 24/10/21 | \| Ingestion of carcass \| \| --- \| |
| 37 | 24/10/21 | Tukum | F | 25 | None | 24/10/21 | \| Ingestion of carcass \| \| --- \| |
| 38 | 24/10/21 | Tukum | F | 35 | None | 24/10/21 | \| Ingestion of carcass \| \| --- \| |
| 39 | 24/10/21 | Tukum | M | 32 | None | 24/10/21 | \| Ingestion of carcass \| \| --- \| |
| 40 | 24/10/21 | Tukum | F | 63 | None | 24/10/21 | \| Ingestion of carcass \| \| --- \| |
| 41 | 24/10/21 | Tukum | F | 17 | None | 24/10/21 | \| Ingestion of carcass \| \| --- \| |
| 42 | 24/10/21 | Tukum | F | 68 | None | 24/10/21 | \| Ingestion of carcass \| \| --- \| |
| 43 | 24/10/21 | Tukum | F | 65 | None | 24/10/21 | \| Ingestion of carcass \| \| --- \| |
| 44 | 24/10/21 | Tukum | F | 65 | None | 24/10/21 | \| Ingestion of carcass \| \| --- \| |
| 45 | 24/10/21 | Tukum | F | 32 | None | 24/10/21 | \| Ingestion of carcass \| \| --- \| |
| 46 | 24/10/21 | Tukum | F | 16 | None | 24/10/21 | \| Ingestion of carcass \| \| --- \| |
| 47 | 24/10/21 | Tukum | M | 63 | None | 24/10/21 | Ingestion of carcass |
| 48 | 24/10/21 | Tukum | M | 14 | None | 24/10/21 | \| Ingestion of carcass \| \| --- \| |
| 49 | 24/10/21 | Tukum | F | 14 | None | 24/10/21 | \| Ingestion of carcass \| \| --- \| |
| 50 | 24/10/21 | Tukum | M | 16 | None | 24/10/21 | \| Ingestion of carcass \| \| --- \| |
| 51 | 24/10/21 | Tukum | F | 45 | None | 24/10/21 | \| Ingestion of carcass \| \| --- \| |
